# From sequencing to validation: NGS-based exploration of plasma miRNA in papillary thyroid carcinoma

**DOI:** 10.3389/fonc.2024.1410110

**Published:** 2024-08-07

**Authors:** WangPeng Cui, Tao Xuan, Tian Liao, Yu Wang

**Affiliations:** ^1^ Department of Head and Neck Surgery, Fudan University Shanghai Cancer Center, Fudan University, Shanghai, China; ^2^ Department of Oncology, Shanghai Medical College, Fudan University, Shanghai, China; ^3^ Runan Medical Technology (Suzhou) Co., LTD, Suzhou, China; ^4^ Shanghai Runan Medical Technology Co., LTD, Shanghai, China

**Keywords:** papillary thyroid cancer, circulating biomarkers, microRNA, cancer diagnosis, NGS

## Abstract

**Objective:**

A non-invasive method using plasma microRNAs provides new insights into thyroid cancer diagnosis. The objective of this study was to discover potential circulating biomarkers of papillary thyroid carcinoma (PTC) through the analysis of plasma miRNAs using next-generation sequencing (NGS).

**Methods:**

Plasma miRNAs were isolated from peripheral blood samples collected from healthy individuals, patients diagnosed with PTC, and those with benign thyroid nodules. The Illumina NovaSeq 6000 platform was employed to establish the miRNA expression profiles. Candidate miRNAs for diagnostic purposes were identified utilizing the Random Forest (RF) algorithm. The selected miRNAs were subsequently validated in an independent validation set using RT-qPCR.

**Results:**

NGS results revealed consistent plasma miRNA expression patterns among healthy individuals and patients with benign thyroid nodules in the discovery set (6 healthy cases, 17 benign cases), while differing significantly from those observed in the PTC group (17 PTC cases). Seven miRNAs exhibiting significant expression differences were identified and utilized to construct an RF classifier. Receiver operating characteristic (ROC) analysis for PTC diagnosis, and the area under the curve (AUC) was 0.978. Subsequent KEGG and GO analyses of the target genes associated with these 7 miRNAs highlighted pathways relevant to tumors and the cell cycle. Independent validation through RT-qPCR in a separate cohort (15 CONTROL, 15 PTC groups) underscored hsa-miR-301a-3p and hsa-miR-195-5p as promising candidates for PTC diagnosis.

**Conclusion:**

In conclusion, our study established a seven-miRNA panel in plasma by Random Forest algorithm with significant performance in discriminating PTC from healthy or benign group. *hsa-miR-301a-3p*, *hsa-miR-195-5p* in plasma have potential for further study in the diagnosis of PTC in Asian ethnic.

## 1 Introduction

There has been a dramatic increase in the incidence of thyroid cancer worldwide in recent years (1). And it has become the 9th most common cancer among all cancers in 2020 (2). The most common type of thyroid cancer is papillary thyroid cancer (PTC), which accounts for about 80% of all cases (3).

For the diagnosis of papillary thyroid cancer, clinical management procedure includes medical history, physical examination, laboratory tests (such as thyrotropin and thyroid hormones), ultrasound, and fine-needle aspiration biopsy (FNAB) (4). Among these, FNAB is the most important assessment tool due to its accuracy, safety, and low cost. However, as an invasive diagnostic method, FNAB carries several complication risks for patients (5). Common complications range from mild issues such as localized pain, discomfort, and hemorrhage or hematomas, to more severe problems like temporary recurrent laryngeal nerve palsy and vasovagal reactions, with some literature even reporting the possibility of needle track seeding of papillary carcinoma (6–8). Additionally, about 20% of thyroid nodules remain indeterminate after FNAB examination, requiring molecular testing for auxiliary diagnosis (9).

Recently, plasma miRNAs have emerged as promising diagnostic molecular markers due to their non-invasive nature, stability and diagnostic sensitivity. miRNAs are small noncoding RNAs encoded by the genome. miRNAs can target multiple mRNAs through complementary sequences, leading to degradation or translation inhibition and ultimately regulating gene or protein expression (10). In cancer, miRNAs function as regulatory molecules, acting as either oncogenes or tumor suppressors. They can promote tumor formation or progression by downregulating tumor suppressor genes, acting as oncogenes. Conversely, they can function as tumor suppressors by downregulating oncogenic proteins (11). miRNAs present are not only in tumor tissues but also in serum and plasma, and the value of circulating miRNAs as diagnostic biomarkers for thyroid tumors has been reported in multiple studies (12–14). In contrast to serum, plasma harbors elevated levels and increased diversity of miRNAs (15), thus presenting heightened potential as a diagnostic biomarker.

The integration of next-generation sequencing and bioinformatics has been widely used in identifying novel tumor biomarkers. Recently, the utilization of machine learning has emerged as a powerful tool in the identification of tumor biomarkers, with the Random Forest (RF) classification model being one of the commonly used methods (16–18). The RF classification model has remarkable advantages in tumor diagnosis, as it can identify the optimal diagnostic panels (19, 20). Currently, several studies have used the RF classification model to identify diagnostic or prognostic panels for various cancers, including thyroid cancer (21–23). However, there are only few reports on the use of NGS technology for studying the miRNA expression profile of PTC plasma miRNAs and their potential for clinical diagnosis. This research gap indicates the need for further studies to explore the diagnostic potential of plasma miRNAs in thyroid cancer patients.

In this study, we aimed to profile the plasma miRNA expression of PTC and compare it with that of benign thyroid nodule patients and healthy population to explore potential diagnostic molecular markers in plasma. A random forest classifier was constructed and 7 miRNAs was selected to form the panel. RT-qPCR were then performed to determine their diagnostic performance in patients with PTC.

## 2 Materials and methods

### 2.1 Patients and samples

This study was approved by the institutional review board of Fudan University Shanghai Cancer Center (FUSCC), and informed consent was obtained from all individuals included in the study.

This study contains 2 sets (**Table 1**). The Discovery set contains 40 samples for sequencing and training the classifier. The Validation set contains 30 samples for RT-qPCR validation. Blood samples and clinical information of potentially eligible participants who were undergoing treatment at our institution (FUSCC) were collected within three days prior to the surgery. The criteria for the eligible participants were pathology-confirmed cases of PTC (defined by the International Classification of Diseases for Oncology 10th Revision as code C73) or benign thyroid nodules (code D34). Potentially eligible participants were selected based on preoperative ultrasound and FNA results of the patients. All patients were pathologically confirmed by postoperative paraffin pathology. Peripheral blood from 6 healthy people (Healthy group) was provided by Shanghai Runan Medical Technology Corporation.

**Table 1:** Sample sizes of three sets.

	Discovery set (N = 40)			Validation set (N = 30)		
Group	Healthy	Benign	PTC	Healthy (CONTROL)	Benign (CONTROL)	PTC
*Sample size*	6	17	17	3	12	15

PTC, papillary thyroid carcinoma.

### 2.2 RNA isolation

Peripheral blood was collected in EDTA Vacutainer tubes (BD Diagnostics, NJ, USA) and processed within 3 h. Plasma was separated by centrifugation at 1,600 × g for 10 min, transferred to microcentrifuge tubes, and centrifuged at 16,000 × g for 10 min to remove remaining cell debris.

Plasma microRNA was isolated using the miRNeasy Serum/Plasma Kit (Qiagen, Hilden, Germany). QIAzol Lysis Reagent was added to the plasma samples. After the addition of chloroform, the lysate was separated into aqueous and organic phases by centrifugation. RNA partitions to the upper, aqueous phase, while DNA partitions to the interphase and proteins to the lower, organic phase or the interphase. The upper, aqueous phase is extracted, and ethanol is added to provide appropriate binding conditions for all RNA molecules from approximately 18 nucleotides (nt) upward. The sample is then applied to the RNeasy MinElute spin column, where the total RNA binds to the membrane and phenol and other contaminants are efficiently washed away. High-quality RNA was then eluted in a small volume of RNase-free water.

### 2.3 Construction and high-throughput sequencing of the microRNA library

According to the protocol of the QIAseq miRNA Library Kit (QIAGEN). Specially designed 3’ and 5’ adapters are ligated to mature miRNAs. The ligated miRNAs are then reverse transcribed to cDNA using a reverse transcription (RT) primer with a UMI. Following cDNA cleanup, library amplification was performed with a universal forward primer and indexing reverse primers. miRNA sequencing libraries were quantified by an Agilent 2100 bioanalyzer and sequenced on an Illumina NovaSeq 6000 platform.

Raw Illumina sequencing reads reflect the total unfiltered reads obtained from the Illumina NovaSeq 6000 platform. Reads initially were filtered for (1) high quality (Illumina “chastity” filter), (2) the presence of the 3′ adaptor sequence (to ensure a small RNA was ligated and sequenced completely), and (3) size of small RNA reads (18–40 nt). Low complexity reads (>50% homopolymer) were removed. These filtering steps resulted in high-quality, filtered reads representing small RNA sequences of 18–40 nt. Then clean unique reads were mapped against all annotated human mature miRNA sequences [miRBase v19.0 (24)] using the program Bowtie (25) with 1 base mismatch at most.

Differential expression analysis was performed with the edgeR (26) Bioconductor statistical library version 3.0.8 on R version 2.15.3. The calculated Counts Per Million (CPM) mapped reads were normalized to log2 (CPM+1), and the resulting data was used for subsequent plotting and analysis (27). After estimating the tagwise dispersion, the gene wise exact test as implemented in edgeR was used to measure the significance of differential expression, using the gene “Pseudocounts”.

Novel miRNAs were predicted using the miRCat tool in the sRNA Toolkit software package (28). miRNAs with False Discovery Rate (FDR, the adjust P-value) ≤ 0.05 and |log2FoldChange| ≥ 2 obtained from edgeR were considered differentially expressed between the two selected groups. The FDR, which is the q-value, was obtained by multiple hypothesis testing correction of the P-value (29, 30).

### 2.4 Random forest classification model

Random forest (RF) classification model is a commonly used and powerful machine learning algorithm with very good performance in classification problems, which was developed by Breiman in 2001 (17). In this study, a RF classification model was constructed to help distinguish the CONTROL group and the PTC group. The RF model was constructed in the R software by the R package “randomForest” with the configuration of “ntree” = {500} (the number of trees to build) (16). The primary RF model was constructed using all the differential miRNAs obtained in differential analysis. The number of selected differential miRNAs was determined based on the results of 5 repetitions of 10-fold cross-validation method. In addition, appropriate miRNAs were selected for further model construction based on the Mean Decrease Accuracy and Mean Decrease Gini obtained from model and the FDR value, |log2FoldChange| obtained from differential analysis.

### 2.5 Validation by real-time qPCR

Total RNA was isolated from samples using the Universal microRNA Purification Kit (EZBioscience, USA). For miRNA analysis, reverse transcription reaction was performed via the microRNA Reverse Transcription Kit PLUS (EZBioscience) according to the manufacturer’s instructions. qPCR was performed using EZ-Probe qPCR Master Mix (EZBioscience) on the QuantStudio™ 7 Flex (Applied Biosystems). U6 RNA was detected as an internal reference. The primer sequences used are listed in **Supplementary Material 1**. The reverse primers for all miRNAs were provided in microRNA Reverse Transcription Kit PLUS. Relative miRNA-expression levels were measured using the 2^–ΔΔCT^ method for each miRNA.

### 2.6 Statistical and bioinformatics analyses

The correlation coefficients and principal component analysis (PCA) were utilized to confirm the biological replicates of the samples (31). The unsupervised hierarchical clustering heatmap of differentially expressed miRNAs (DEmiRs) across all samples was generated using the “Pheatmap” package in R software. Receiver operating characteristic (ROC) curves and areas under the curve (AUCs) were used to evaluate the diagnostic performance. MiRanda (32) was used to predict target genes of DEmiRs (including known and novel miRNAs), followed by Gene Ontology (GO) and Kyoto Encyclopedia of Genes and Genomes (KEGG) pathway enrichment analysis. In addition to R, the programs used for plotting and analysis included GraphPad Prism software, version 9.0.0 (GraphPad Software, San Diego, CA, United States) and SPSS version 25.

## 3 Results

### 3.1 Characterization and NGS analysis of the discovery cohort

The clinicopathological characteristics of the 40 samples from the discovery set were recorded in detail (**Table 2**). Importantly, there were no significant differences in the distribution of patients among the Healthy, Benign, and PTC groups in terms of age, sex, lymphocytic thyroiditis and ethnicity, ensuring the data balanced and comparable. Across the discovery set, the majority of patients were under the age of 55, with 6 (100%), 14 (82.4%), and 13 (76.5%) individuals in the Healthy, benign, and PTC groups respectively. Gender distribution was found to be balanced across the groups, with 3 (50%), 10 (58.8%) and 9 (52.9%) individuals in the Healthy, benign, and PTC groups respectively. All patients in the discovery set belonged to the Asian ethnic group. Furthermore, differences in distribution were observed in tumor multifocality and size between the Benign and PTC groups. Multifocality was more prevalent in the benign group (14, 82.4%) compared to the PTC group (2, 11.8%), demonstrating a statistically significant difference (p < 0.001). Similarly, maximum tumor less than 2 cm were more frequently observed in the benign group (13, 76.5%) than in the PTC group (2, 11.8%), also displaying statistical significance (p < 0.001). This balanced distribution of clinical characteristics minimizes errors in subsequent comparisons, while variations in tumor multifocality and size introduce potential sources of error.

**Table 2:** Clinicopathological information of discovery set.

Variable	Healthy group		Benign group		PTC group		P-value
*Age*, *No. (%)*							0.427
*<55*	6	(100)	14	(82.4)	13	(76.5)	
*≥55*	0	(0)	3	(17.6)	4	(23.5)	
*Gender*, *No. (%)*							0.909
*Male*	3	(50)	10	(58.8)	9	(52.9)	
*Female*	3	(50)	7	(41.2)	8	(47.1)	
*Lymphocytic thyroiditis*, *No. (%)*							0.831
*Yes*	0	(0)	3	(17.6)	2	(11.8)	
*No*	6	(100)	14	(82.4)	15	(88.2)	
*Multifocal*							<0.001
*Yes*	/	/	14	(82.4)	2	(11.8)	
*No*	/	/	3	(17.6)	15	(88.2)	
*Maximum tumor/nodule size*, *No. (%)*							<0.001
*<2 cm*	/	/	13	(76.5)	2	(11.8)	
*≥2 cm*	/	/	4	(23.5)	15	(88.2)	
*Ethnicity*, *No. (%)*							1
*Asian*	6	(100)	17	(100)	17	(100)	

**Figure 1:**
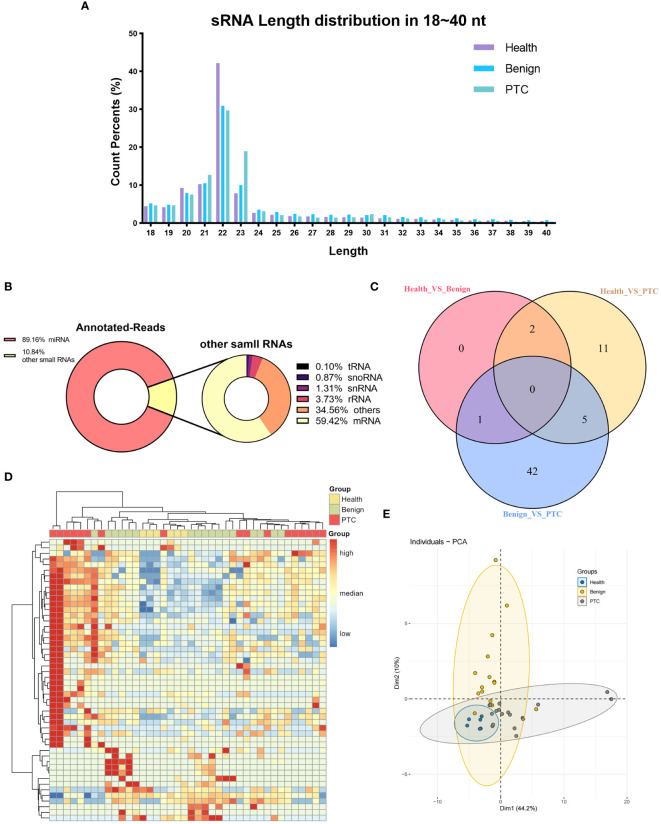
Characterization of small RNA sequencing data. **(A)** The percentage of small RNAs with a length ranging from 18 ~ 40 nt. **(B)** Annotated-reads distribution of small RNAs. The left diagram illustrates the annotation status of annotated small RNAs ranging from 18 to 40 nt; the right diagram shows the annotation status of other small RNAs depicted in the left diagram. **(C)** Venn diagram of differentially expressed miRNAs in three differential analyses (Healthy VS Benign, Healthy VS PTC, Benign VS PTC). **(D, E)** Clustering analysis **(D)** and principal component analysis **(E)** of all 50 known miRNAs of significant miRNAs that were differentially expressed in three differential analyses (Healthy VS Benign, Healthy VS PTC, Benign VS PTC).

NGS sequencing was then performed on all samples from the discovery set. Raw reads were obtained by sequencing from 40 samples in Discovery set. Subsequently, clean reads were obtained after filtering low-quality reads. Only clean reads of small RNAs with lengths in the range of 18-40 nt (total 309,45 million) were utilized for subsequent analysis. The results showed that small RNAs were mainly distributed in the vicinity of 20-23 nt, and the peak length of distribution was 22 nt (**Figure 1A**), which was consistent with the typical length of miRNAs. Based on the comparison with the reference genome (GRCh38) and annotation by miRBase, small RNAs were categorized into miRNAs, rRNAs, tRNAs, snoRNAs, snRNAs, mRNAs, and others. A total of 89.16% of the small RNAs were annotated as miRNAs, showing a good quality of library construction (**Figure 1B**). Of the other small RNAs that accounted for 10.84% of the total, mRNAs (59.42%) were predominantly present, with others such as rRNAs (3.73%), snRNAs (1.31%), snoRNAs (0.87%), and tRNAs (0.10%).

In addition, reads of small RNAs that were not annotated to any known RNAs were used for the prediction of novel miRNAs. The prediction of novel miRNAs relied on the signature hairpin structure of the miRNA precursor. A total number of 569 Novel miRNAs were predicted by miRCat software (**Supplementary Material 2**).

We subsequently conducted an analysis of annotated miRNAs and novel miRNAs to assess their differential expression across the healthy, benign, and PTC groups. From this analysis, a total of 61 DEmiRs were identified through comparisons among the groups (healthy vs. benign, benign vs. PTC, healthy vs. PTC) (**Figure 1C**). Notably, the DEmiRs consistently showed upregulation in both comparisons involving the PTC group (healthy vs. PTC and benign vs. PTC). Specifically, miRNAs such as *hsa-miR-145-5p*, *hsa-miR-424-5p*, *hsa-miR-1-3p*, *hsa-miR-223-3p*, and *hsa-miR-152-3p* were found to be upregulated in the PTC group compared to both healthy and benign groups.

Furthermore, there were only three DEmiRs between the Healthy and Benign groups (**Supplementary Material 3**), suggesting that the Healthy and Benign groups shared similar miRNA expression patterns. And these three miRNAs also showed deregulation when these two groups were compared with the PTC group. Among them, the expression of *hsa-miR-219a-2-3p* was up-regulated in the Benign group than in the Healthy group; and its expression was also up-regulated in the PTC group than in the Benign group, suggesting that the same cut-off could be used to separate the PTC group from the Healthy and Benign groups.

Using all the known miRNAs of DEmiRs detected above, clustering analysis were performed to assign the Discovery set plasma samples into groups with similar miRNA expression patterns (**Figure 1D**). Interestingly, clustering analysis showed that Healthy and Benign group shared common miRNA expression profiles. And the principal component analysis (PCA) also showed that the Healthy samples were included in the Benign samples (**Figure 1E**).

The aim of this study was to identify a panel of miRNAs capable of distinguishing PTC samples from both healthy and benign samples. Given the similarity in miRNA expression patterns between the Healthy and Benign groups in plasma, combining these samples into a single group (CONTROL group) may facilitate the identification of significant DEmiRNAs.

### 3.2 Identification of differential miRNAs and classification model

**Figure 2:**
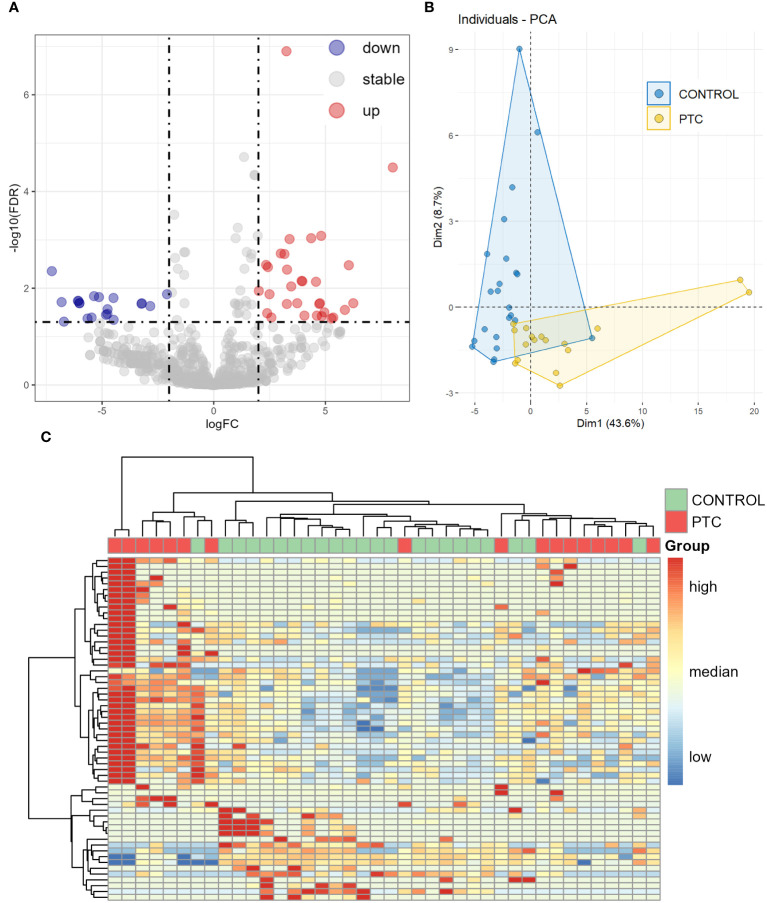
Differentially expressed miRNAs in plasma between CONTROL and PTC samples. **(A)** Volcano diagram of Differentially expressed miRNAs in plasma between CONTROL and PTC group. miRNAs with False Discovery Rate (FDR) ≤ 0.05 and |log2FoldChange| ≥ 2 were considered significant. **(B, C)** Principal component analysis **(B)** and clustering analysis **(C)** of all 59 known miRNAs that were differentially expressed in four differential analyses (Healthy VS Benign, Healthy VS PTC, Benign VS PTC, CONTROL VS PTC).

We then analyzed the differential expression of the miRNAs between the CONTROL and PTC groups. Analysis of plasma samples between the CONTROL and PTC groups revealed a total of 51 DEmiRs by the criteria of False Discovery Rate (FDR, the adjusted P-value) ≤ 0.05 and |log2FoldChange| ≥ 2 (**Figure 2A**). The **Table 3** showed some of the miRNAs with significant expression differences (List of all DEmiRs in the analysis of the CONTROL vs PTC groups is provided in **Supplementary Material 4**).

**Table 3:** Top down/up-regulated miRNAs of PTC group in CONTROL vs. PTC comparison.

Down-regulated			Up-regulated		
miRNAs	Log_2_FC	FDR	miRNAs	Log_2_FC	FDR
***hsa-miR-516b-5p***	-7.25	4.43E-03	***hsa-miR-199b-5p***	3.47	9.16E-03
***hsa-miR-92b-3p***	-2.10	1.33E-02	***hsa-miR-424-5p***	3.39	9.63E-04
***novel.247***	-5.36	1.46E-02	***hsa-miR-199a-5p***	3.28	4.11E-03
***novel.128***	-5.14	1.53E-02	***hsa-miR-411-5p***	3.27	2.11E-02
***hsa-miR-550a-3p***	-4.49	1.59E-02	***hsa-miR-223-3p***	3.25	1.26E-07
***hsa-miR-517a-3p***	-6.08	1.83E-02	***hsa-miR-145-5p***	3.17	1.96E-03
***hsa-miR-517b-3p***	-6.06	1.83E-02	***hsa-miR-206***	3.00	1.91E-03
***novel.269***	-6.81	1.93E-02	***hsa-miR-3157-3p***	2.58	4.04E-02
***novel.111***	-6.01	2.04E-02	***hsa-miR-184***	2.50	1.33E-02
***novel.41***	-6.01	2.04E-02	***hsa-miR-152-3p***	2.43	3.66E-03
***hsa-miR-6734-5p***	-3.22	2.04E-02	***hsa-miR-196a-5p***	2.39	3.29E-02
***hsa-miR-1247-5p***	-3.24	2.10E-02	***hsa-miR-18a-3p***	2.34	3.30E-03
***hsa-miR-195-5p***	-2.84	2.34E-02	***hsa-miR-301a-3p***	2.02	1.13E-02

MiRNAs, micro RNAs; Log_2_FC, log base 2 of the fold change; FDR, false discovery rate (the adjusted P-value).

Including all known miRNAs of DEmiRs from the results of the four previous comparisons for principal component analysis (PCA) and unsupervised clustering analysis, the CONTROL and PTC samples clustered into different categories, as expected that Healthy and Benign samples had similar expression patterns (**Figures 2B, C**).

**Figure 3:**
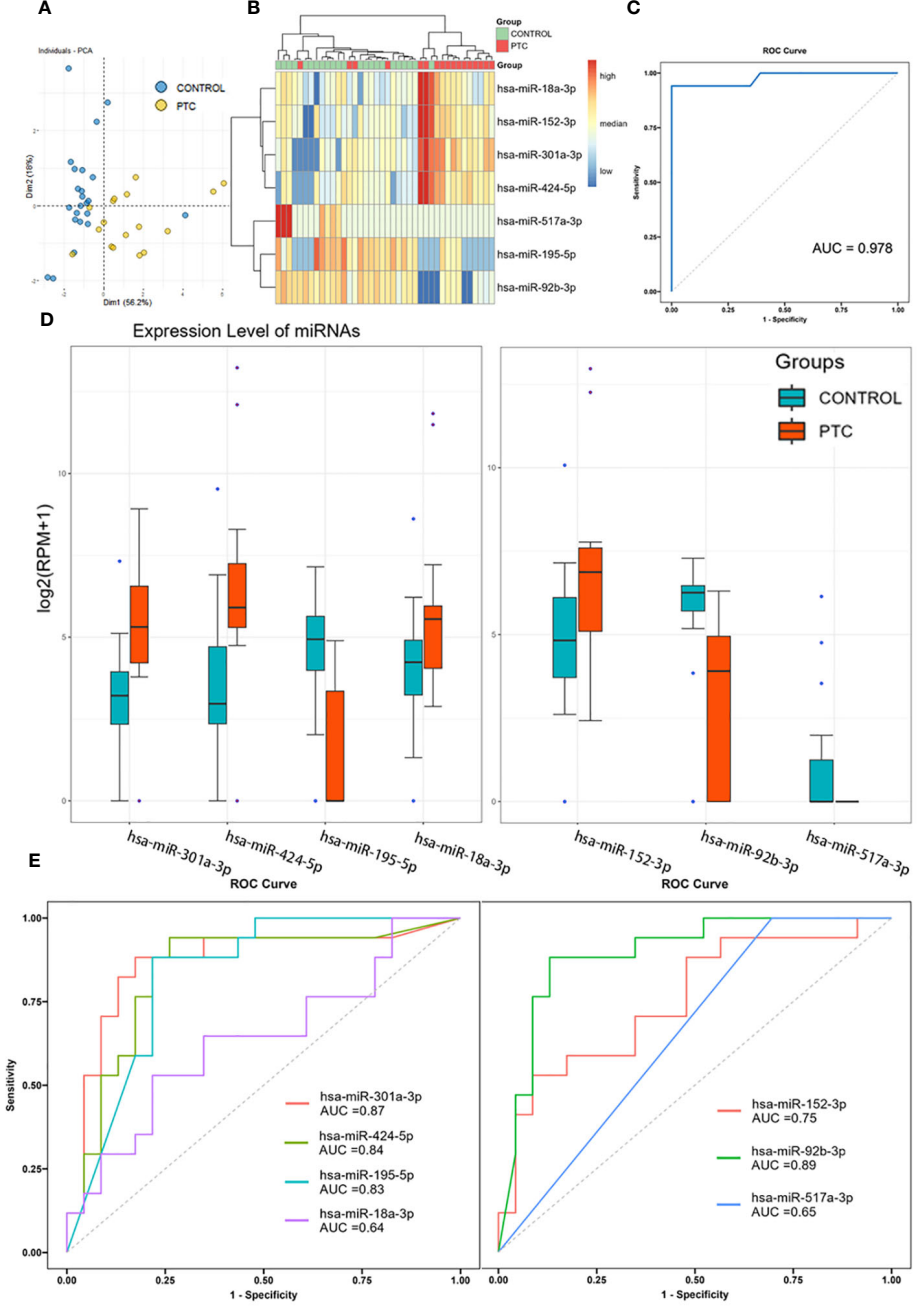
Diagnostic accuracy of the 7 miRNAs panel. **(A, B)** Principal component analysis **(A)** and clustering analysis **(B)** of 7 miRNAs identified by Random Forest algorithm. **(C)** Receiver operating characteristic analysis and area under curve of the 7-miRNAs Random Forest classifier in distinguishing PTC from CONTROL patients. **(D)** Expression level of 7 identified miRNAs in Discovery set. **(E)** Receiver operating characteristic analysis and area under curve of the 7 miRNAs when used to distinguish PTC from CONTROL patients alone.

To further screening, the Random Forest (RF) algorithm was used to construct a classifier to distinguish PTC from CONTROL. After two step-by-step screenings and final debugging, a panel of 7 miRNAs were identified from all known DEmiRs using the Random Forest algorithm. The 7 miRNAs panel is a powerful predictor used to distinguish PTC from CONTROL patients (including *hsa-miR-301a-3p*, *hsa-miR-424-5p*, *hsa-miR-18a-3p*, *hsa-miR-195-5p*, *hsa-miR-152-3p*, *hsa-miR-92b-3p*, *hsa-miR-517a-3p*). Principal component analysis was performed using these 7 miRNAs, with the first two principal components explaining 74.3% of the total variance and the first three principal components explaining 85.7% of the total variance. The diagram of PCA showed that the majority of the CONTROL and PTC group samples were well separated (**Figure 3A**). In addition, unsupervised cluster analyses also showed that the CONTROL group samples were well clustered, with only 1 sample incorrectly clustered to the PTC group (**Figure 3B**). Receiver operating characteristic (ROC) analysis further demonstrated the sensitivity and specificity of this 7-miRNAs panel in distinguishing PTC from CONTROL patients. The area under curve (AUC) of this panel reached an astonishing 0.978 (**Figure 3C**). In addition, to verify the specificity of the classifier in thyroid cancer, we tested it using 3 negative control sets consisting of patients with other types of cancer from an external database, which also had good detection results (**Supplementary Material 5**). In contrast, when the expression levels of the 7 miRNAs were compared individually, reduced differences were found between the two groups (**Figure 3D**). And when these 7 miRNAs were used to distinguish the two groups of patients alone, the AUC was only 0.64-0.89, much lower than that of the 7 miRNAs panel (**Figure 3E**).

### 3.3 Target gene prediction and pathway enrichment analysis

**Figure 4:**
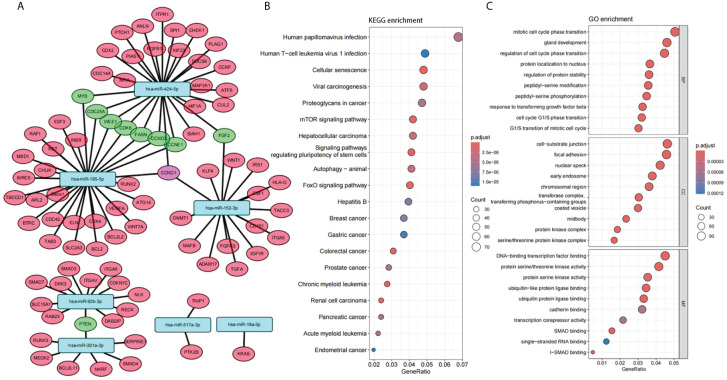
Function of the panel of 7 miRNAs. **(A)** Interaction network between 7 selected miRNAs and their target genes. **(B, C)** Enrichment analysis of Gene Ontology (GO) categories **(B)** and Kyoto Encyclopedia of Genes and Genomes (KEGG) pathways **(C)** for target genes of 7 selected miRNAs.

In order to gain further insight into the possible role of these 7 miRNAs in PTC, target gene prediction was performed for these 7 miRNAs. There were ten genes co-targeted by more than two miRNAs, one of which was co-targeted by three miRNAs (CCND1), and another nine genes were co-targeted by two miRNAs (**Figure 4A**). Nine of these 10 genes are closely associated with tumor development (CCND1, MYB, CDC25A, WEE1, CDK6, CCND3, CCNE1, FGF2, PTEN) and 6 of them are mainly associated with the cell cycle (CCND1, CDC25A, WEE1, CDK6, CCND3, CCNE1).

Enrichment analysis of Gene Ontology (GO) categories and Kyoto Encyclopedia of Genes and Genomes (KEGG) pathways for these target genes also confirmed the association with tumor and cell cycle (**Figures 4B, C**). Several tumor-related pathways were identified, such as Proteoglycans in cancer, mTOR signaling pathway, FoxO signaling pathway. Cell cycle-related pathways have also been identified, such as Mitotic cell cycle phase transition, Regulation of cell cycle phase transition, Cell cycle G1/S phase transition, G1/S transition of mitotic cell cycle. In addition, several viral carcinogenesis-associated pathways were significantly enriched (Viral carcinogenesis, Human papillomavirus infection, Human T-cell leukemia virus 1 infection, etc.), suggesting that plasma miRNAs may be closely associated with oncogenic viral infection and viral carcinogenesis.

In conclusion, these results suggest that these 7 miRNAs identified in this study can not only be used as molecular markers for the adjuvant diagnosis of PTC, but also have the potential to be used as therapeutic targets for tumor therapy because of their functional relevance to the development of PTC.

### 3.4 Validation of miRNAs in independent validation set by RT-qPCR

**Figure 5:**
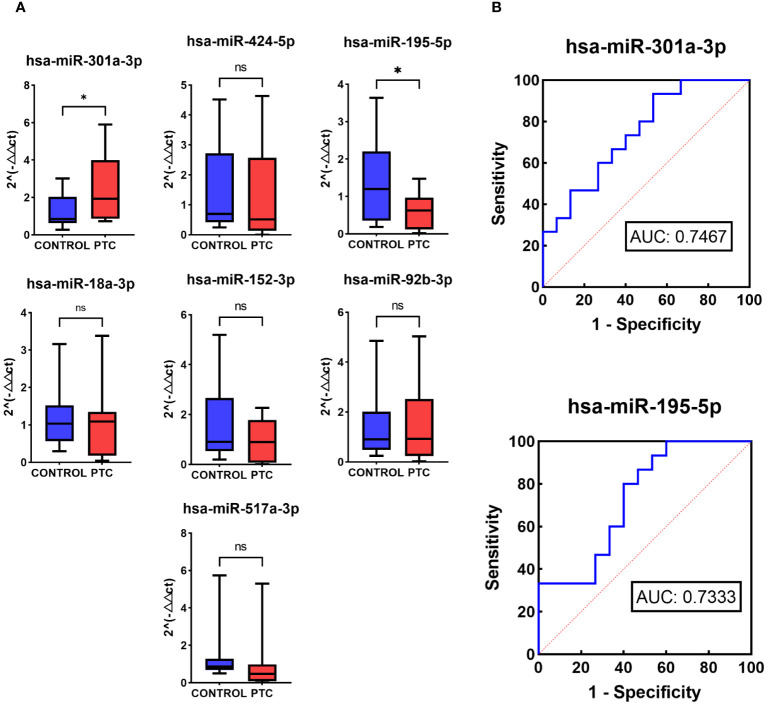
RT-qPCR of 7 selected miRNAs in Validation set. **(A)** Relative expression levels in RT-qPCR of 7 selected miRNAs in the Validation set of the CONTROL and PTC group. **(B)** Receiver operator curves of *hsa-miR-301a-3p* and *hsa-miR-195-5p* in validation set for diagnosis of PTC from CONTROL group. The symbol ‘*’ means p<0.05, i.e. the difference is statistically significant. The symbol ‘ns’ means P>0.05, which means that the difference cannot be proved to be statistically significant.

To validate the performance of the 7 selected miRNAs, RT-qPCR was conducted using a new, independent Validation set comprising 15 CONTROL samples and 15 PTC samples (**Table 1**). Among these, 3 out of the 7 miRNAs exhibited consistent expression trends as observed in the NGS results (*hsa-miR-301a-3p, hsa-miR-195-5p, hsa-miR-517a-3p*). Specifically, *hsa-miR-301a-3p* showed up-regulation in the PTC group, whereas *hsa-miR-195-5p* and *hsa-miR-517a-3p* were down-regulated in the PTC group. Moreover, statistical analysis indicated that two of these three miRNAs, *hsa-miR-301a-3p* (P = 0.0226) and *hsa-miR-195-5p* (P = 0.0147), displayed statistically significant differences in expression between the PTC and CONTROL groups (**Figure 5A**).

To further evaluate the diagnostic performance of *hsa-miR-301a-3p* and *hsa-miR-195-5p* in diagnosing PTC, ROC analysis was performed. ROC analysis showed that the two miRNAs had a potential ability to discriminate PTC from CONTROL with AUCs of 0.7467 and 0.7333 (**Figure 5B**).

## 4 Discussion

Thyroid nodule is a common thyroid disease in the population, some of which may be malignant. Although FNA combined with cytopathology is the gold standard method for diagnosis of malignant thyroid nodules, there are still some tested thyroid nodules remain indeterminate, which brings biomarkers as an adjunctive diagnostic method (33). In this study, a panel of seven plasma miRNAs identified by the Random Forest algorithm was found to have potential for the diagnosis of papillary thyroid carcinoma. In the initial discovery set, this study analyzed the plasma miRNA expression profiles of healthy controls, patients with benign thyroid nodules and patients with PTC. Interestingly, we observed a consistent plasma miRNA expression profile between samples from the healthy and benign groups. To enhance the analysis of miRNA differential expression in the PTC group, we amalgamated the healthy and benign groups into a single CONTROL group. Our analysis revealed significant differences in the plasma miRNA expression profile of the PTC group compared to the CONTROL group. An initial RF classifier including all identified differential miRNAs was then constructed, and after debugging, a 7-miRNAs classifier with high diagnostic performance was finally constructed. This RF classifier showed high performance in diagnosing PTC with an AUC of 0.978. Further RT-qPCR was performed in an independent validation set and identified 2 miRNAs with significant difference (*hsa-miR-301a-3p*, *hsa-miR-195-5p*). Subsequent analysis enriched and examined the functions of the target genes regulated by these 7 miRNAs, revealing their involvement in various tumor and cell cycle-related pathways. Thus, these miRNAs not only serve as promising non-invasive biomarkers for diagnosing PTC patients but also present potential as therapeutic targets.

In recent years, with the development of sequencing technology and the emerging advantages of miRNAs as non-invasive diagnostic biomarkers, several studies have focused on circulating miRNAs for the diagnosis of PTC. A genome-wide study of plasma miRNAs using RNA array found that *miR-25-3p* and *miR-451a* may be of value in the diagnosis of PTC (34). A study of plasma miRNAs from healthy individuals, benign thyroid nodules and patients with PTC using the Exiqon panel found that a panel of 3 miRNAs consisting of *miR-346*, *miR-34a-5p* and *miR-10a-5p* could be used for the diagnosis of PTC (35). Many researchers have highlighted that *miR-146b* may have potential for the diagnosis of PTC (36–38). On the other hand, some researchers have investigated miRNAs in exosomes. *miR-16-2-3p* and *miR-223-5p* are exosome miRNAs that can be used to discriminate between PTC and benign thyroid nodules, identified by studying isolated plasma exosomes using RNA sequencing (RNA-seq) technology (39). A study including both plasma and serum found that exosome *miR-485-3p* and *miR-4433a-5p* could be used for PTC diagnosis, and *miR-485-3p* could also be used to discriminate between high-risk and low-risk PTC (13). Although there have been some studies focusing on circulating miRNAs, there are still large differences in the types of miRNAs reported by different researchers, as well as relatively small sample sizes and difficulties in validation, so further studies on the role of plasma miRNAs in PTC screening are still needed.

For the plasma miRNAs identified in this study, there have been previous studies suggesting possible roles in tumors. Many studies have found that *hsa-miR-195-5p* is expressed at low levels in a variety of tumor tissues, including lung cancer (40, 41) and breast cancer (42). It has been shown that *hsa-miR-195-5p* can inhibit the role of cell proliferation in thyroid tumors by inhibiting cyclin D1, which is a potential therapeutic target for thyroid tumors (43). In contrast, *hsa-miR-301a-3p* tends to be overexpressed in tumor tissues and shows a role in promoting tumor growth (44, 45). In clear cell renal cell carcinoma (ccRCC), *hsa-miR-301a-3p* is associated with potential metastatic potential, and this study also suggests that the function of plasma exosome miRNAs may be related to tumor cycle, proliferation, etc. (46).

The diagnostic panel constructed in this study includes five other miRNAs, which also play roles in the diagnosis or prognosis of various cancers. *hsa-miR-424-5p* may play an important role in the pathogenesis of thyroid cancer and cholangiocarcinoma (47, 48). Additionally, *hsa-miR-424-5p* also holds potential as a diagnostic and prognostic marker in gastric cancer, laryngeal squamous cell carcinoma, colorectal cancer, and liver cancer (49–55). In breast cancer, *hsa-miR-424-5p*, in combination with another miRNA, *hsa-miR-142-3p*, can be used to inhibit tumor cell proliferation (56). *hsa-miR-18a-3p* plays a central role as one of the hub miRNAs in the ceRNA networks involved in hepatocellular carcinoma and adult T-cell leukemia/lymphoma (57–59). Furthermore, exosomal *hsa-miR-18a-3p* has the potential to serve as a biomarker to distinguish between breast cancer and breast fibroadenoma (60). *hsa-miR-152-3p* not only acts as an anti-tumor miRNA in thyroid cancer by negatively regulating ERBB3 but is also significantly associated with hypertension-related RCC (61–63). Additionally, *hsa-miR-152-3p* may inhibit pathway activation by negatively regulating PIK3CA expression, thereby suppressing cell proliferation and functioning as a tumor suppressor in human breast cancer cells (64). *hsa-miR-92b-3p* and its targets may promote PCA metastasis through platinum resistance and the JAK-STAT signaling pathway (65). Furthermore, *hsa-miR-92b-3p* is related to the overall survival of patients with liver cancer (66). *hsa-miR-517a-3p* is associated with liver cancer and the pathways involved in cancer and tumorigenesis (67).

In our study, the target genes of the panel of 7 miRNAs we identified are primarily associated with tumor progression. These genes include CCND1, MYB, CDC25A, WEE1, CDK6, CCND3, CCNE1, FGF2, and PTEN, among others, with nine genes being targeted by more than two miRNAs. Additionally, six of these genes (CCND1, CDC25A, WEE1, CDK6, CCND3, CCNE1) have been shown in multiple studies to be related to the cell cycle (68–73). Besides its association with the cell cycle, WEE1 is also involved in DNA repair in tumors (74, 75). Enrichment analysis of the target genes identified several tumor-related pathways, including Proteoglycans in cancer, the mTOR signaling pathway, and the FoxO signaling pathway. Cell cycle-related pathways were also identified, such as Mitotic cell cycle phase transition, Regulation of cell cycle phase transition, Cell cycle G1/S phase transition, and G1/S transition of the mitotic cell cycle. Additionally, several pathways associated with viral carcinogenesis were significantly enriched, including Viral carcinogenesis, Human papillomavirus infection, and Human T-cell leukemia virus 1 infection. These findings suggest that plasma miRNAs may be closely associated with oncogenic viral infection and viral carcinogenesis.

This study has several limitations that warrant consideration. Firstly, the cohort primarily consisted of Chinese patients, limiting generalizability to individuals of diverse ethnicities, lifestyles, and clinical characteristics. This may affect the reproducibility of findings when validating using external datasets. Secondly, the sample size in this study remains relatively small, necessitating inclusion of a larger number of samples in future studies to robustly validate the diagnostic efficacy of the identified miRNA panel. Lastly, while the target genes of the miRNAs have been predicted and functionally enriched, their precise roles in tumor development require further exploration and validation.

## Data availability statement

The datasets presented in this study can be found in online repositories. The names of the repository/repositories and accession number(s) can be found in the article/**Supplementary Material (Table 5)**.

## Ethics statement

The studies involving humans were approved by the institutional review board of Fudan University Shanghai Cancer Center, Fudan University Shanghai Cancer Center, Shanghai. The studies were conducted in accordance with the local legislation and institutional requirements. The participants provided their written informed consent to participate in this study.

## Author contributions

WC: Formal analysis, Investigation, Validation, Visualization, Writing – original draft. TX: Conceptualization, Formal analysis, Visualization, Writing – review & editing. TL: Conceptualization, Writing – review & editing. YW: Conceptualization, Resources, Supervision, Writing – review & editing.
