# Supplementary material for: From sequencing to validation: NGS-based exploration of plasma miRNA in papillary thyroid carcinoma
Source: Front Oncol. 2024 Aug 7;14:1410110. doi: 10.3389/fonc.2024.1410110 (PMC11335555; doi:10.3389/fonc.2024.1410110)
Supplement: Supplementary file 1 [file DataSheet_1.docx]

**Supplementary material 5** Diagnostic accuracy of the constructed classifier in negative control cohorts.

To validate the specificity of the classifier for thyroid cancer, the accuracy was tested using three sets of patients with other types of cancers from the GDC database as negative controls. We used part of the data from GSE210329 (breast cancer), GSE244605 (liver cancer), and GSE240757 (lung cancer) to form the three negative control cohorts.

The results showed that the classifier still showed good diagnostic performance in the three negative control sets. The classifier showed an AUC of 0.957 (breast cancer set), 0.968 (liver cancer set), and 0.968 (lung cancer set) in negative control sets.

**Table** Sample sizes of three negative control sets.

| Group | Negative control set 1 | | | |  | Negative control set 2 | | | |  | Negative control set 3 | | | |
| --- | --- | --- | --- | --- | --- | --- | --- | --- | --- | --- | --- | --- | --- | --- |
|  | *Health* | *Benign* | *Breast Cancer* | *PTC* |  | *Health* | *Benign* | *Liver Cancer* | *PTC* |  | *Health* | *Benign* | *Lung Cancer* | *PTC* |
| Sample size | 6 | 17 | 8 | 17 |  | 6 | 17 | 9 | 17 |  | 6 | 17 | 9 | 17 |


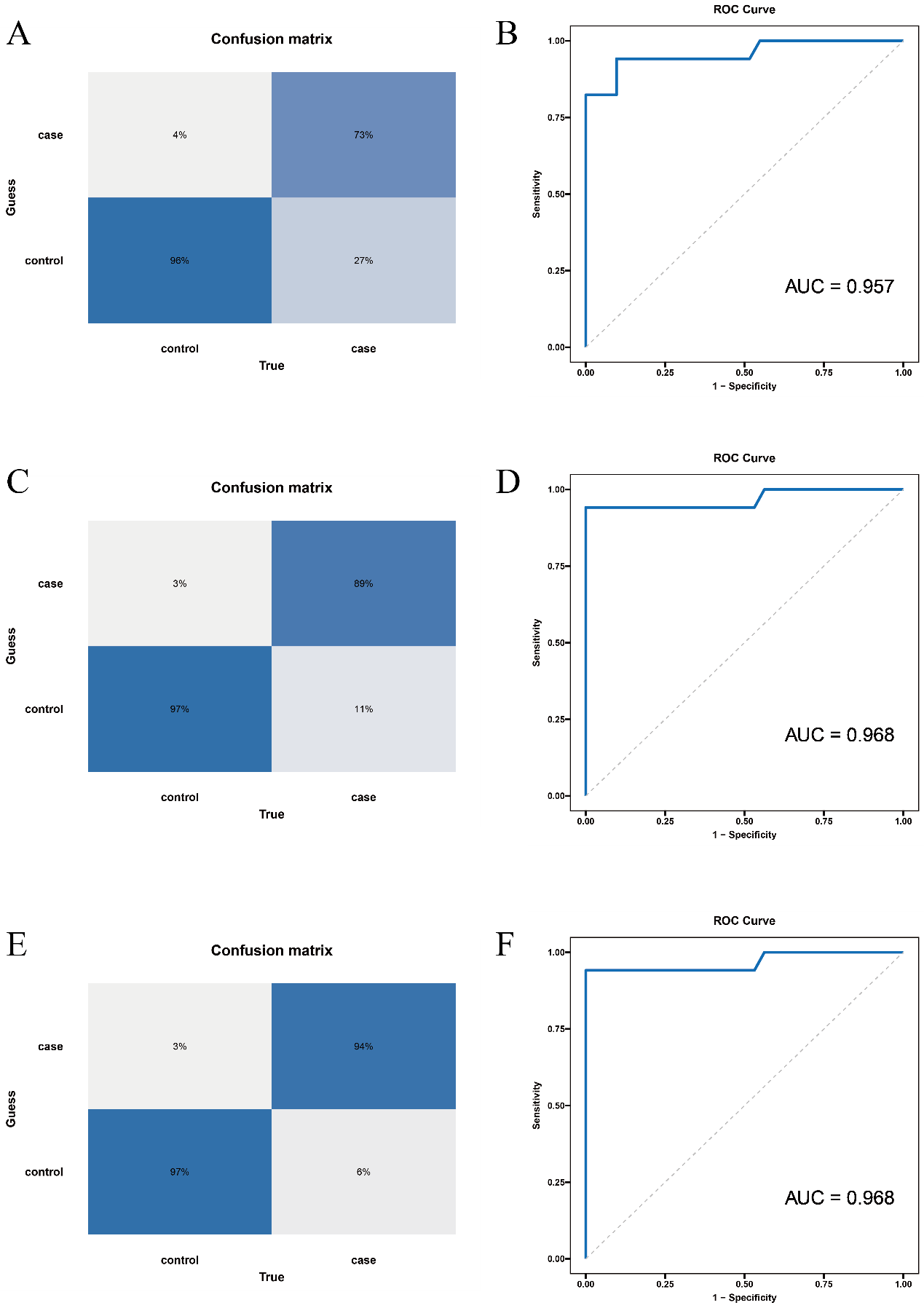


**Figure** Diagnostic accuracy of the constructed classifier in negative control sets. (**A**) and (**B**) Confusion matrix and ROC curve of negative control cohorts with breast cancer. (**C**) and (**D**) Confusion matrix and ROC curve of negative control cohorts with liver cancer. (**E**) and (**F**) Confusion matrix and ROC curve of negative control cohorts with lung cancer.
