# Supplementary material for: From sequencing to validation: NGS-based exploration of plasma miRNA in papillary thyroid carcinoma
Source: Front Oncol. 2024 Aug 7;14:1410110. doi: 10.3389/fonc.2024.1410110 (PMC11335555; doi:10.3389/fonc.2024.1410110)
Supplement: Supplementary file 2 [file Table_1.docx]

**Supplementary material 1** Primers used for RT-qPCR

| miRNA | Forward primer | | |  |
| --- | --- | --- | --- | --- |
| U6 | 5'- | CCTGCTTCGGCAGCACA | -3' | |
| hsa-miR-301a-3p | 5'- | GAGGGCGAATAGTAATGTCAAAGG | -3' | |
| hsa-miR-424-5p | 5'- | GGGCAGCAGCAAGTCATGTT | -3' | |
| hsa-miR-18a-3p | 5'- | GGTGCTGCCCTAAGTGCTCC | -3' | |
| hsa-miR-195-5p | 5'- | GGTTAGCAGCACAGAAATAGGG | -3' | |
| hsa-miR-152-3p | 5'- | GGGCTATGACAGAACTTGGCG | -3' | |
| hsa-miR-92b-3p | 5'- | GTGCTATTGCTCTCGTCCCG | -3' | |
| hsa-miR-517a-3p | 5'- | ATCGTGCTTCCCTTTAGAGTGTG | -3' | |

The reverse primers for all miRNAs were provided in microRNA Reverse Transcription Kit PLUS.
