# Supplementary material for: From sequencing to validation: NGS-based exploration of plasma miRNA in papillary thyroid carcinoma
Source: Front Oncol. 2024 Aug 7;14:1410110. doi: 10.3389/fonc.2024.1410110 (PMC11335555; doi:10.3389/fonc.2024.1410110)
Supplement: Supplementary file 3 [file Table_2.docx]

**Supplementary material 2** Sequences of novel miRNAs

>novel.1

GCACGGTTACGAGAGCAGG

>novel.2

AGCAAGGAAGAGGATTGGAAG

>novel.3

GTGAAGATGCGGAGTACC

>novel.4

TCGGCGGGCGCGGGGGCGG

>novel.5

CTACAGAACCGCAGAGGCCT

>novel.6

TGGCTCTGAGGGCTGGGT

>novel.7

GGAGAAGCCGGCGGGAGC

>novel.8

CAACACCGTCTCCGGCCC

>novel.9

TTGCACTTGGAGAACACTTG

>novel.10

CCGGAGGAAGGTGGGGAC

>novel.11

TGGAGTTGAGTTTGCAGC

>novel.12

AAAAGTAGTTGCGGTTTTTG

>novel.13

TCCCAGTCTGGCCTGAGTTTTGC

>novel.14

CCATTGGCTGTGGAGTCGG

>novel.15

TGAGACTGGGGAGAAAGG

>novel.16

AGTAGAGTTGGGAGTTCG

>novel.17

TGAGTGAACTTTTTAAAA

>novel.18

ATCATGTAGAAGTAAAACCAAAT

>novel.19

TGCGGGCCGTGAGGGGTGT

>novel.20

TGCGGGCCGTGAGGGGTGT

>novel.21

TGCGGGCCGTGAGGGGTGT

>novel.22

TGCGGGCCGTGAGGGGTGT

>novel.23

TGCGGGCCGTGAGGGGTGT

>novel.24

TGGATGGGATCGGAGCTCT

>novel.25

GGGGGCGGGCTCCGGCGG

>novel.26

CGTCTTTGCAGAGTGCTG

>novel.27

AAGGGAGTGTCTGTAATGG

>novel.28

TCACCCCTGCTTCCTCTGCCAGA

>novel.29

AGCCGAGGGAAGGAAGAATA

>novel.30

TTCCAAGCAGTTGACCCGGG

>novel.31

AGCAGAGCCAGAATGTGAAT

>novel.32

GTAGAGGGAATTTCCAGTGG

>novel.33

AGAAAGGATATTTCAGAA

>novel.34

GAGTGAAGCTGGGGACCTT

>novel.35

TTCAGAGATGAGATTGTG

>novel.36

CAGGAGCTGGCTGAGGAG

>novel.37

TCCAGACTTAGGAAGGATT

>novel.38

TGCGGGGTGGGAAGAGGAGA

>novel.39

CTTATCTCTCAGTTGCCC

>novel.40

TTTTTGGTTACTGTTTGA

>novel.41

GGAGAGGTGGATGAGTGG

>novel.42

AGTTCGAGTCTTGCCTGGG

>novel.43

AAGAGACTGTTTTTTTTT

>novel.44

CAAAGGCAGAAGGGAGCT

>novel.45

GGACAAGAAGGGAAGGGTAGAG

>novel.46

TCAGACGTCTCGAGGCTCGCG

>novel.47

AGTCCATTGGATTGAGTT

>novel.48

AGCCGGGGGAAGTTGAGG

>novel.49

AAAAGTTATTGCGGTTTTGG

>novel.50

CAAAAGTAATGGCGGTTTTT

>novel.51

ACAAGGCAAGAAGATCAC

>novel.52

CCTGGACTGTCTCGGCCCTGT

>novel.53

GGAGGGATGGGTGAGTGG

>novel.54

TTAGTGACTTTTCCAAACT

>novel.55

GCATGATTTTGAGGGTTC

>novel.56

GGTAGAATTCTCGCCTGC

>novel.57

TCGGGGCGGCGGCGGCGG

>novel.58

GGGAAGAGGAGGAGAGGG

>novel.59

TCGCCCAAAGTCACACAAGTTA

>novel.60

GGGCGGCGGCGGTCGGCGG

>novel.61

CACTGTTTTATAAAGACT

>novel.62

CACCCCTTCCCTCAGCTCCTGA

>novel.63

GCCCTGTGGACTCAGTTC

>novel.64

CGGGGCTGGGAGTGGAGAGG

>novel.65

AGATCTTGGTGGTAGTAG

>novel.66

TCCCTCTAAGAAGTCCAC

>novel.67

GCTTGGGAGGTTGGGACA

>novel.68

CGTGGTGGCCCGTGCCTGTGG

>novel.69

ATGAATGGATGAATGAGA

>novel.70

CAAATGCAGATTCCTGGACTCCA

>novel.71

TGTGCAGGTGCTGTGTGG

>novel.72

GGGATTCCTGGCTCTTGG

>novel.73

CCAACACAGGAGGCACTGACCA

>novel.74

CTGTGGGGACATTTGTGG

>novel.75

GGCTGGGCTGGGCTGAGGCT

>novel.76

AGTAAGGTGGATTGTGAT

>novel.77

CTTATGCCCATGTCTGGAGTCGG

>novel.78

GAGAGATACCAGAGAGCTCT

>novel.79

AAGAGAAAGTCCAGAGGC

>novel.80

CAGTGCAATGTTAAAAGG

>novel.81

CCTCAGACCACAACCCCATG

>novel.82

AGGCCGGAGTGCAGTAGTGTGA

>novel.83

GCGGGGCTGGCTCTGGGCCT

>novel.84

CCACTCGGCCAGGAATGC

>novel.85

AAAAGTAATTGAGGTTTTGG

>novel.86

TGAGCGGCCGGGAGGATTTA

>novel.87

CAACTAGACTGTGAGCTTCT

>novel.88

CAACTAGACTGTGAGCTTCT

>novel.89

GGAGTTTGGCTGGGGTGG

>novel.90

CTCACTGTAGCCTCGAACCCCT

>novel.91

AATTGAGGTTTTATCTGAGGGGA

>novel.92

ATCCCACCACTGCCACCA

>novel.93

CCACTGCAACCTCTGCCTCTT

>novel.94

GGTTCCAGCAGCTCCCCTCA

>novel.95

TTCACCTGACCAAACAAA

>novel.96

CTCTGAGGGAGGAGGAAG

>novel.97

CCCCTGCCTTTCCTCTATAACT

>novel.98

AAGAGCACAGGGGTCCAGGCA

>novel.99

GATGCTGAGATGCTGGAGG

>novel.100

GTGGGAGAGGAAGGTAGA

>novel.101

TTGGTGTGTGTCTGTGTC

>novel.102

GGGCGGCGCGGCGGCGGCG

>novel.103

CAAAAGTAATGGCGGTTTTT

>novel.104

GGAAAAGAAAAAGGAGGG

>novel.105

CCTGTTTGAGTGTCTGTT

>novel.106

TGGGGAAGATGAGGAAGG

>novel.107

CTGGAGGGAGGGAGGAAGG

>novel.108

GTGTGGGTTTGATTTGTTGT

>novel.109

CATTCTGCTTCTTAGAGG

>novel.110

GCTGGGCTGGGCTGAGGCT

>novel.111

GGAGAGGTGGATGAGTGG

>novel.112

CATCTCAAATCTCAAATC

>novel.113

GTGGATCCACAGTGCAAA

>novel.114

AAAAGTAGTTGCGGTTTTT

>novel.115

ACAGTGGCTGGACCTTTG

>novel.116

TTGGGGTTTGGGGTTTGG

>novel.117

TTTTATCTGTTGTTTGTT

>novel.118

ATGGATATCTAGGCTCCT

>novel.119

GCAATGAATGAGAGCTCCA

>novel.120

CGAGGTGAGGATTTGGAG

>novel.121

GGGAGGGCGGCGCCGCCG

>novel.122

GGGGAGTTTGGCTGGGGC

>novel.123

CGGGGGCGGCGCGCGCGCG

>novel.124

TGCGAGTGGTTTTCTGAGGAGT

>novel.125

CTTGAGTATGAGAGAGGT

>novel.126

CCTCAGATTCTGGATTTCTACC

>novel.127

TGTTGTACTTTTTTTTTTG

>novel.128

TTCCTCTGCTCTCATACCACA

>novel.129

TGACTTGATTTTGATTTT

>novel.130

GTGACCACTGTGACTATG

>novel.131

CCTGTGGATTTACCTGCA

>novel.132

AAGTAGGAGGAGGAAGAG

>novel.133

TTCCATTGGTATTTTAGG

>novel.134

GATTCCTGTACTTGTTTG

>novel.135

TGTGATGCTGCTGGCCTG

>novel.136

AAACAACAGAAATTAACAA

>novel.137

GGTCTTGAGAATGTTTTG

>novel.138

CGGGGAGGGTGGGTGAAGAGC

>novel.139

GAGGGAGATGGTTTCGGG

>novel.140

CCATGTGATTTCCAGTCA

>novel.141

TTGCACTTGGAGAACACTTG

>novel.142

TTATACTTGGGAGCTTTTCT

>novel.143

TCAGGTAGTAGGGCATTGTGGTT

>novel.144

CGGCGCGCCGGGCCCGGG

>novel.145

GATGTTGCTGGTGTTGGT

>novel.146

CGATTGCGCCACTACCCT

>novel.147

TAGGCAGGCGTGGAGGTG

>novel.148

GTTGGGGAGAATGTGAGG

>novel.149

TGAGATTTGAGAGGGGCTG

>novel.150

GTGAAATGTTTAGGACCA

>novel.151

CTGCAGCACCTCCAGCTCCA

>novel.152

GTGGCCGCTGACGGGCGGGG

>novel.153

CCCAGTTCAGCTCAGCTCAGC

>novel.154

CCCAGTTCAGCTCAGCTCAGC

>novel.155

CCCAGTTCAGCTCAGCTCAGC

>novel.156

AGGGCAGGGCGCCCTGGA

>novel.157

TGACTGATATTTTCGCTA

>novel.158

GGCAGGGGCCGGCGGCGG

>novel.159

CTTGATTTCTTTTCCTAC

>novel.160

TCCCTGTCCTCCAGGAGCT

>novel.161

TGTGGTCTAGTGGTTAGG

>novel.162

TTGGCGGTCGATGGGACC

>novel.163

AACAGTAATTGTGGTTTTTGC

>novel.164

AGGAAGGGAGAAAAGAGA

>novel.165

TTCCTCTTCTTAACACCA

>novel.166

AGAAGAAGAGGAGGAGGAT

>novel.167

CGCTCATGAGTCTCTGTT

>novel.168

TTGGGAATGCTGCTCTAA

>novel.169

GGGGAGCTGGGCAGAAGG

>novel.170

CTTGGGACTGGCTCAGAG

>novel.171

TGCTGGGGAGAGAGGAGG

>novel.172

AGAGGATAGATAGATAGA

>novel.173

GGGGATATAGGAGCCATCA

>novel.174

TAGGTGGCTGGAGTGAGGGTG

>novel.175

GCCGGGCGCGGTGGCGCGCG

>novel.176

CCCAGCTTGAGTTTTTCTGTTGG

>novel.177

CTGCAGACTCGACCTCCCAGG

>novel.178

TGTGTTGTGTGTGGGGTG

>novel.179

CCTGGACTGTCTCGGCCCTGT

>novel.180

TGGTGCACTGGCTGTGGA

>novel.181

CAGCACCCAGTGTTCCCA

>novel.182

CGCCCCCGCCGCCGGGGCC

>novel.183

TTAGATGTGTATATAATG

>novel.184

TGGCTCTCAGTGTGGGGA

>novel.185

CTGGCCCTCTCTGCCCTT

>novel.186

CTGGGCTGGGCTGAGGCT

>novel.187

CCGCCCCCCGGCCCCGCG

>novel.188

TTGAGGGCCGGCCGTGGAAG

>novel.189

CAGGACGGGGATTTGAAC

>novel.190

GGCCCATCCAGCCCCACA

>novel.191

CGACGACGCTCAGGCCCGGCC

>novel.192

AAAGTAATTGTGGTTTTTGCTG

>novel.193

TCGACGCCCGGCTGCCAGC

>novel.194

CCGCTCCGGCCCGCGCGG

>novel.195

GGGAAGAGGAGGAGAGGG

>novel.196

AGGGAGAAAGAAAGAGGG

>novel.197

GTGTGAGGCAATAACAGG

>novel.198

CCTGGCTGGGTTTATGGAGCCT

>novel.199

GCGGCAGTGAGGCTGTGG

>novel.200

AAAAGTTATTGCGGTTTTGG

>novel.201

CTCCTCGCCACGCCCCGCCCTT

>novel.202

AGGGAGTGGGGGAGGGTGGGAA

>novel.203

AATGGCTCTTCTCAGTCGGTGA

>novel.204

ACATGCGCCCTCGGCTTCTGG

>novel.205

TGGGGAGAGAGGATGTGG

>novel.206

TTAGGTAGTTGGTAAGGT

>novel.207

ACCGTTGAAGTATTTTTG

>novel.208

ACTCCAACTTTCCTTCCT

>novel.209

GGGAAGAGGAGGAGAGGG

>novel.210

CACATTTCCCCCTTTTTGG

>novel.211

TAGGCCTTCTCTGATTGGA

>novel.212

TGGTAGGGAGCTAGGACTTT

>novel.213

CCAGTTGAGATAAGTCTC

>novel.214

GTGCCAGAGGAGGAAGTG

>novel.215

TTGGGCTTGCTCTTTGCA

>novel.216

AACAGAATAATACTGGAA

>novel.217

GGAGTTTGGCTGGGGTGG

>novel.218

TTCTCTCTGTCTTTCTCTCTCA

>novel.219

GGCGGCGGCGGCGGGGGTG

>novel.220

TTTGTCTGGGGCCTGGTGAGT

>novel.221

TGGAGTGTGACAATGGTG

>novel.222

TTTTTCTTTTCTTTTGTCT

>novel.223

ACGGGGAGGAAGGGAGGAT

>novel.224

AACAGTAATTGTGGTTTTTGC

>novel.225

AGGGGGTCATGGAAAAAGGT

>novel.226

AAGGGACGGAAGGGCAAG

>novel.227

AGGGGTGGGTATTGTAGA

>novel.228

GGCGCGGGGCGCGGGGCGG

>novel.229

CGGCGGCGGGCGCGGGGGG

>novel.230

TGCTGGGGAGAGAGGAGG

>novel.231

CTCCCCCTCCCCTCCCCG

>novel.232

ATCTCCCGCCTCCTTTTCCCG

>novel.233

TCTGGGAAGAAGCTGTGGGAC

>novel.234

TGTTCCTGCTGAACTGAGCCAG

>novel.235

TAAGCTACTGTGCCTGGCCAGT

>novel.236

AGACGTCACGGGAGAGGGGCA

>novel.237

TGCGCCTGACCTGCTCCTG

>novel.238

CCTTCCTCAAGTCTCCAAC

>novel.239

GGCGGGTGCGGGGGTGGG

>novel.240

CCAATCCTTCAGATCCTCCTCA

>novel.241

GATGTGGAGTTTGGCTGGG

>novel.242

GGTCCCGCGCTAGTCCCAC

>novel.243

ATTTTGTTGGTTTTTTGT

>novel.244

AGCTCAATGAGGACAGAAA

>novel.245

AAAGTAATTGTGGTTTTTGCTG

>novel.246

AAAGTAATTGTGGTTTTTGCTG

>novel.247

TTGCAGCTGCCTGGGAGTG

>novel.248

TATTTTCCTGCTGGTTGG

>novel.249

AGAGGTCGAGGCAGCAGTGAG

>novel.250

CAACTGGGTTCTGGGGGG

>novel.251

ATGGGTTGTATGTTACATGCATG

>novel.252

AGTGAAGCTCAACAGGGT

>novel.253

CCAGGGCAAATGTTTGTACTCC

>novel.254

TGGGGCTGGATGAGGGGCAGG

>novel.255

CGGCAGTGAGGCTTGGGG

>novel.256

CTTTTGATCTCCCTTGCT

>novel.257

TCTAAGAAGATCAGCCAG

>novel.258

CCCGGTGAGGCGGGGGGG

>novel.259

GGCTGGTTGGCTGGTTTG

>novel.260

ACTGAAATTCATATTGTT

>novel.261

GAATCTGGCTGGAACAGT

>novel.262

ACTGAAATTCATATTGTT

>novel.263

GGCTGGTTGGCTGGTTTG

>novel.264

GCCCGGCGGGCGCCGGCG

>novel.265

TCAGACTACCTAAATGAGCACT

>novel.266

CAGTGTGCTTTGCACAAG

>novel.267

TTTGGGGTTTGGGGTTTGG

>novel.268

TGCCTGTTTGAGTGTCTGTT

>novel.269

ATTTTGTGGGTGGTGGTG

>novel.270

CCTTCACGCCCTCGGGCT

>novel.271

TTCTACATGAGATTTCTG

>novel.272

GGCGGGCGGCGGGGCGGGG

>novel.273

CTCAGGCCTGGTTAGTAG

>novel.274

TGAGCACACATGTTGGGA

>novel.275

ACTTAGAGGCTTTCAGTA

>novel.276

TTTTTCAGATTTTGGAATGTTTG

>novel.277

GGCGGCGGTGGTGGTGGTG

>novel.278

GTTTTTCTTCATCTTCCT

>novel.279

TGGTAGTGTGGCTCAAGC

>novel.280

CAAGCTGCTCTCCAAGAA

>novel.281

ATATTGAAGAGAAAGGGA

>novel.282

CGCGGCGGCGGCGGGGGG

>novel.283

ACCATATAGATATCCACACA

>novel.284

TTTGTTTTGTTGGTTTTAATG

>novel.285

TGCTTTTCAACTTTCCCT

>novel.286

AATTCCACTGACCTCTCC

>novel.287

TTTGAAGTGAGAGGTGTC

>novel.288

CGGCAGTGAGGCTTGGGG

>novel.289

AGCAATCACAGCCCAACCACC

>novel.290

TGGTGGCTCGCATCTGTGGT

>novel.291

TGTTCTGCTCCATGGTCGTTCA

>novel.292

TAGAGGAGAGAAGGAAATG

>novel.293

TCTCTTTTCCTGGGCTTGTGGG

>novel.294

TGGCAGAAGACATGAAACT

>novel.295

TCTGTGGCTGTGTGTGTG

>novel.296

TCTGTGACCTGGGGCAAGTG

>novel.297

CCTCACATCCCAGAACTT

>novel.298

CCCCGGGGAGCCCGGCGG

>novel.299

TGGCTCTGAGGGCTGGGT

>novel.300

TCGCTCTTTGTCTCGGCTCG

>novel.301

TGAGGCACGACTTGGGATGAGA

>novel.302

GTAGGAGTGCCCGTCGGGACG

>novel.303

GTAGGAGTGCCCGTCGGGACG

>novel.304

TAGGCCTTCTCTGATTGGA

>novel.305

TAGGCCTTCTCTGATTGGA

>novel.306

CCTCCAACGCCTTTTCAA

>novel.307

CTCCGTCCCCTCTCTCCCTTC

>novel.308

AAGGAGAATCAAAGCTGA

>novel.309

CTGCCACTGTTGCTCTTG

>novel.310

GAGATTTGAGAGGGGCTG

>novel.311

ATGGGTTGTATGTTACATGCATG

>novel.312

CATCCCCGCCTTCCTCCTG

>novel.313

CTACTGGGTTTGTGCCTG

>novel.314

CACGTGTACAGCCCTGGGCA

>novel.315

GAGATTTGAGAGGGGCTG

>novel.316

TAGGCCTTCTCTGATTGGA

>novel.317

CCTGGACTGTCTCGGCCCTGT

>novel.318

GGCTCTCCCGGTCCCTGCTGG

>novel.319

TGTGCAGGTGCTGTGTGG

>novel.320

TGTGGGCAATTGGGAAGG

>novel.321

TATGGGGGATACCTTGGC

>novel.322

GTTGTTATTTGGAGTTTT

>novel.323

CCGTCTTTCCTCGTCTTGCCCG

>novel.324

CGACCGCGTCCGCCAGGCCG

>novel.325

AGCGCGTCCCGCGGGAGAGGTG

>novel.326

GGCGGGCGGCGGGGCGGGG

>novel.327

TGTGGGGAGATTAAAGGA

>novel.328

CTTCCTCTCTCTTTCTTG

>novel.329

AGGGGATCATGGAGGGTGA

>novel.330

TTTCCTCTGCTCTCATACC

>novel.331

TCCATTACACTACCCTGCCTCTT

>novel.332

CGCCCCCGGGGCCGCGGTT

>novel.333

GTTGATTGCAGAGTGGTG

>novel.334

TCAGATTCTAGTCTTCCCTAAA

>novel.335

GACTGGTTTTGTCGGGGG

>novel.336

TTCGTTCCCGGAGTGGCTGGCG

>novel.337

GCGGGTGCGGGGGTGGGC

>novel.338

CCTTCAGGCATTGGCCTGTG

>novel.339

TGAGATTTGAGAGGGGCTG

>novel.340

CAGGCTTTCACCTCTGACT

>novel.341

AACAGTAATTGTGGTTTTTGC

>novel.342

CTTATCTTCAGTATATAA

>novel.343

GTTATCCAGAGTACCTTT

>novel.344

AACCAAGTCCAAGTCTGG

>novel.345

TGCCCTGAGACTTTTGCT

>novel.346

AACAGTAATTGTGGTTTTTGC

>novel.347

TGCTGGGGAGAGAGGAGG

>novel.348

CGAATTAAAGCCACTGATA

>novel.349

GTGCAAAAGTAATGGCGGT

>novel.350

AAAAGTAGTTGCGGTTTTTG

>novel.351

GTTTTGCTGATCTTTTGC

>novel.352

TGTGTTGTTTGTTTTTTGG

>novel.353

AGCTGGTTTCTTCTGAAG

>novel.354

CGGGCCAGTGGCGGAAGGG

>novel.355

TCCTCATCTGTACGGGGGGA

>novel.356

CTCCCTCTACCTCACTGT

>novel.357

GTGCGATGGAAGGGACTC

>novel.358

CTTTGGGTCGCGGGGGCG

>novel.359

TAAAGCTGGCAACAATAAGGCC

>novel.360

GAGGGGACTTGGTACAGG

>novel.361

ATATGGTTTGGATGTTGT

>novel.362

GTCCTTAAGTGATTTTGG

>novel.363

TGGGAGAGAGTGGAAAGC

>novel.364

CTTGTCAATCATTTTCTA

>novel.365

TGTTGGTTTCTAGGAATG

>novel.366

TTTCAGAGTGGAAGCATTG

>novel.367

AAACAGGATAGGCACTAAATGG

>novel.368

AACAATGAGGGGAAAACT

>novel.369

TGAGATTTGAGAGGGGCTG

>novel.370

CAAAAGTAGTTGCGGTTTTTG

>novel.371

CCGAGCGGCTGCGCGCGGG

>novel.372

GGCGGGGTTGTGGGAGAG

>novel.373

CAAGAGCAGTTGGTTGAT

>novel.374

GCGGGCCATCACTGTTGGAGG

>novel.375

AACAGTAATTGTGGTTTTTGC

>novel.376

GTCCAGATTGGAGTCTGA

>novel.377

AAAAGTAATTGAGGTTTTGG

>novel.378

TACTCAAGAGGCTGAAGAG

>novel.379

CGGCGGGCCATCACTGTTGGAG

>novel.380

CAGTTTTGGAAAGGTAGG

>novel.381

GTCACACCCTGGGAATGG

>novel.382

TCAGTGAAATTGTAATGG

>novel.383

AAAAGTAGTTGCGGTTTTTG

>novel.384

CAGTAACTCCCCTCCGCCCCTG

>novel.385

CGGCGGGCCATCACTGTTGGAG

>novel.386

GCGGGCCATCACTGTTGGAGG

>novel.387

CGGCGGGCCATCACTGTTGGAG

>novel.388

GCGGGCCATCACTGTTGGAGG

>novel.389

CGGCGGGCCATCACTGTTGGAG

>novel.390

GCGGGCCATCACTGTTGGAGG

>novel.391

CGGCGGGCCATCACTGTTGGAG

>novel.392

AGCAGCCCTTGCATCTCAAC

>novel.393

CACTGACAATTCAATGGT

>novel.394

CCTCCTCCTCATCGTCATC

>novel.395

CAGCAGATTCAGTAGTTGG

>novel.396

TTTATTAGCAGCAAAACT

>novel.397

ATTTTCTTTGTTTTTGCTG

>novel.398

TGTAGGGCAAGTCTGGTG

>novel.399

CACACCTGTAGTCCTAGTACTT

>novel.400

TGTGACTCCCCTGTGGCTGGTG

>novel.401

CCCACTGCAACCTCCGCCTCA

>novel.402

GAACTCTTCCCAGGGCCA

>novel.403

TCCGGGGCTGGGAGTGGAGAGGG

>novel.404

ACTCCACACTCAGCCCGGCTGG

>novel.405

TCTCTGTTTTCCGACCTCTCC

>novel.406

GTAAGGAAGAGGTCTTGG

>novel.407

GTCCAGTTTTCCCAGGAAT

>novel.408

CTGGACTTGGAGTCAGAAG

>novel.409

TGGCAGTGAGGCTGTGGG

>novel.410

ATTTTAGAGACGGGGTCTTG

>novel.411

GAAAAGGAGAGAGAAAGG

>novel.412

GTTCGCATTGTTGATCTG

>novel.413

GGGCTCTGGTTCTGGTGT

>novel.414

ACAGCCCAGATCGTCAGC

>novel.415

GTCTTGGGTTGGGGTTGG

>novel.416

TAGGCCTTCTCTGATTGGA

>novel.417

TAGGCCTTCTCTGATTGGA

>novel.418

GGAGAGGAAGAGAAGGGA

>novel.419

TATCTTACTTTGAAACTG

>novel.420

TCAGGATTGGACCTGGGACTGA

>novel.421

GAGAGGTTGAAACAATGA

>novel.422

TGGTTCATTCAAATTTCTG

>novel.423

AGCCCGAGTTCGAGGTTACAGT

>novel.424

GGGAATTAGCTCAAGTGGTAGAG

>novel.425

GGGGGTGTAGCTCAGTGG

>novel.426

GCCTGGATAGCTCAGTTGG

>novel.427

GGGGTATAGCTCAGTGGTAGAG

>novel.428

GTAAGGAGGGGGATGAGG

>novel.429

CTCCCCCGTCTCCGCCCC

>novel.430

GTGGGGGCGGCGGCGGGG

>novel.431

CCCTCCTCCCCGCGCCCC

>novel.432

CCAATGCCACTCTGAAGC

>novel.433

TCTGGGTTGGGAGGCGTGG

>novel.434

AGGTTAGAAGTGGAAGTGT

>novel.435

CCCTCCTCCCCGCGCCCC

>novel.436

TTTTTCAGATTTTGGAATGTTTG

>novel.437

CTCACAGACACACACCCCAC

>novel.438

CTCATCTACCAAGTGACCCA

>novel.439

AGAGGTCGAGGCAGCAGTGAG

>novel.440

TTACTAACATGCAAATTG

>novel.441

GGAGTTTGGCTGGGGTGG

>novel.442

CCACTGCAACCTCTGCCTCTT

>novel.443

GGGGGATCATGCAGGTGAGTGGG

>novel.444

CCGGACTGGCTGTCCATGCTG

>novel.445

AGGGCTGGGTCTAGGGGT

>novel.446

GGTTGTGGGAACGACGGGG

>novel.447

GAGGGGCCCGGGGCGGGG

>novel.448

CCAATGTCGCATGGGGTGTACA

>novel.449

AGGGGAGTTTGGCTGGGG

>novel.450

CAGTTTTGCCAATTGTTGT

>novel.451

TCTGTATCTTTTTCTTTGG

>novel.452

CTGGGAATGAAGTAGAAA

>novel.453

TGTGTTTTGTTTTGGATT

>novel.454

GGCGGCGGTGGTGGTGGC

>novel.455

CTGGAAGAAAAAGGCTTT

>novel.456

GGCGGCGGCGGGGGTGTG

>novel.457

GGGAAGAGCCCAGCGCCGA

>novel.458

CTTGAAATTGTTAAAAGG

>novel.459

TCGTCTCAGAATCACCCA

>novel.460

GGCGCGGGGCGCGGGGCGG

>novel.461

TGGAAAAGAGCATTAGAG

>novel.462

TCTTTCTTTCTGGGGAACT

>novel.463

TTCGAGATGCGCCTGGGCA

>novel.464

GGCTTGGGTTCAGGAGTTCG

>novel.465

GCTGGGCTGGAAGTTGGGG

>novel.466

AAGTAGGAGGCTCTGATG

>novel.467

TTTTCTTGCAAAGTTGGG

>novel.468

GGTGGTGATTTCACTGACA

>novel.469

TCGCTCTAACCGCCGCGCGC

>novel.470

ATATGTAAAAGAACAGTG

>novel.471

GATTCTTCCTTGGGGTGG

>novel.472

GGCCCCGCCCGCCCCTCGCGCA

>novel.473

ACTAGAGAAACTCCAGGG

>novel.474

GGCCTCAGTAGTTCTATTAAA

>novel.475

CAGATCAAGAGGTCCCCGGT

>novel.476

AAACACTCACTCCCAGGGTCA

>novel.477

TGTTGTTGTTATTGTATTAGA

>novel.478

CAGGCTGGAGATGAGTGTC

>novel.479

TTGGGGTTTGGGGTTTGG

>novel.480

CTTCTCCCTTTCCCAGCAA

>novel.481

ATCCCACAGCTGCGTCATATCC

>novel.482

TGCACTGCCAGATCTCGCCAAT

>novel.483

TGTACTGTGCTGTGTGTT

>novel.484

GGTTTGCAGAGTGCTGTG

>novel.485

AAGTGGGAGGAGGAGGAG

>novel.486

GGTGGGGAGTTGGCTGGG

>novel.487

AAGCAGAGGGTCATAGGT

>novel.488

TCATATTACTTCATTTCCCAA

>novel.489

CACAGGTGGTGCATGGCTG

>novel.490

CGCCCACTCCTCCCGCCTCGG

>novel.491

ATGCCTCCCTGTCGCTGCT

>novel.492

CGTTGTTCTAGTTTGAGG

>novel.493

CGTTGTTCTAGTTTGAGG

>novel.494

GCAAAAAGGACACTGGTACC

>novel.495

ACTGAATACATAGGTTGG

>novel.496

AACGGAGGCGCTCAAAGG

>novel.497

CCTAGTCTACTGGAGGATAAG

>novel.498

CTTTTGCCCTTTTGTTCTA

>novel.499

GCCTCCTTAGCGCAGTAGG

>novel.500

AGAAGGAGAGAGAAAAGGA

>novel.501

ACTCCCGGGAGCGGCGCGGT

>novel.502

CAGGAACTGCCCTTCTCTCCA

>novel.503

GAGTGTGGAGTGTGGAAT

>novel.504

GGTGGGGTGGATGAGTGG

>novel.505

CAGCCCGGCCTCCTCCAGTGCA

>novel.506

TTCCCCTATTCCTAAATGTGCT

>novel.507

GATCCCAGCGGGACCCGCG

>novel.508

TTAGGAATGTGGGCTCTGGATTG

>novel.509

CTTTTTGTTTTTGTTGCTT

>novel.510

CTGTATCAGTATTTATTG

>novel.511

GTGTTTGGGCTCTGGGGA

>novel.512

CGGGGCGACGCTCGGGTT

>novel.513

TAGGCCTTCTCTGATTGGA

>novel.514

TAGGCCTTCTCTGATTGGA

>novel.515

CTCAGGCCTGGTTAGTAG

>novel.516

CCCACTGCTAAATTTGACTGGTT

>novel.517

AGAGAGACAAAGTTATAG

>novel.518

TCCTGCGATGGGACGGTGCTG

>novel.519

TGGACTTTATTTTCATGCTGTGG

>novel.520

TGGACTTTATTTTCATGCTGTGG

>novel.521

TCCTGCGATGGGACGGTGCTG

>novel.522

GTCCGGCGGGAGGAGAGAGGC

>novel.523

AGGAGCGCGCGCATGTCAAGG

>novel.524

TTCCTGCTGAACTGAGCCAGT

>novel.525

CTTGTCTGGCTACTGCTGGTGA

>novel.526

GAGGGCGATGAGGGAGGG

>novel.527

AGAGCTGCCTCGGTGCCACA

>novel.528

TCGGCCATGGAAGTAAGT

>novel.529

AGGAAGGGAGGAAACCAGAGG

>novel.530

CTTTTCCATCTCATCTTG

>novel.531

CTTTTTGGATCTGTTGAAA

>novel.532

GAGCTGAGGGTAGGGGTG

>novel.533

AGCAAGCTGTCGGTGCTG

>novel.534

TCCGCTGCCACGCTGTGACCAG

>novel.535

TGAGGCACGACTTGGGATGAG

>novel.536

CACTGTGGCCAGAGAGGGAGGG

>novel.537

CCTGGACTGTCTCGGCCCTGT

>novel.538

GTAGGAGTGCCCGTCGGGACG

>novel.539

TTTCTAGGACCGCTGTAA

>novel.540

TCAGCTTTGGATGTTAGG

>novel.541

CAAAAGTAGTTGCGGTTTTTG

>novel.542

GAGGCCTGGGAAATCTTG

>novel.543

TTTTGTGGTTGGTGTGGG

>novel.544

CTCTTTGACATTCTGTTCC

>novel.545

GTGAGTTTTGTTTGGACA

>novel.546

GGCGGCGGCGCGGACGCG

>novel.547

GTCAGTTTGTCAAATACC

>novel.548

CAAGAAGATTGAGAGGAGT

>novel.549

CTTATCAGATTGTATTGT

>novel.550

GATGAAAAGCACTTTGAA

>novel.551

GCAGATTCCTAGGCTTTAC

>novel.552

CCTGGTCTTTGGAGTGAG

>novel.553

TGTCCTTGGGCCTCTTTGT

>novel.554

TGAACTTGTATTTCTTTC

>novel.555

GGGCATTGAAGAGCTGGG

>novel.556

CTGTCTGGGGTTTTCTGT

>novel.557

GCTTGAGTCTGGGAAGTG

>novel.558

CCCTGTCCCTCCTTGGCT

>novel.559

CATGCTGGGCTCTTCCCCC

>novel.560

GTTTGGATTTGATTTTAA

>novel.561

GTTTTGTTGTTGTTGAAA

>novel.562

CGCTTCCCCCCACTCGCCCTCG

>novel.563

TAGGAACGGTTGGGGACA

>novel.564

CTGTTTGGGCTTTGTTGT

>novel.565

ACATCACCCGGGTGATCAGT

>novel.566

GTACTGGCTAGTTGTGTT

>novel.567

TTGTGTCAGAATCATAAT

>novel.568

TTGTGTCAGAATCATAAT

>novel.569

TTGTGTCAGAATCATAAT
