# Supplementary material for: From sequencing to validation: NGS-based exploration of plasma miRNA in papillary thyroid carcinoma
Source: Front Oncol. 2024 Aug 7;14:1410110. doi: 10.3389/fonc.2024.1410110 (PMC11335555; doi:10.3389/fonc.2024.1410110)
Supplement: Supplementary file 4 [file Table_3.docx]

**Supplementary material 3** differential miRNAs between Health and Benign Groups.

| **Health VS Benign Group** | | |  |
| --- | --- | --- | --- |
| up-regulated |  | down-regulated | |
| *hsa-miR-219a-2-3p* |  | *novel.550* | |
| *hsa-miR-218-5p* |  |  | |
