# Supplementary material for: From sequencing to validation: NGS-based exploration of plasma miRNA in papillary thyroid carcinoma
Source: Front Oncol. 2024 Aug 7;14:1410110. doi: 10.3389/fonc.2024.1410110 (PMC11335555; doi:10.3389/fonc.2024.1410110)
Supplement: Supplementary file 5 [file Table_4.docx]

**Supplementary Material 4** Differential miRNAs between CONTROL and DTC Groups.

| CONTROL VS Benign Group | |  |
| --- | --- | --- |
| down-regulated | **up-regulated** | |
| hsa-miR-516b-5p | *hsa-miR-223-3p* | |
| hsa-miR-92b-3p | *novel.372* | |
| novel.247 | *hsa-miR-542-3p* | |
| novel.128 | *hsa-miR-1-3p* | |
| hsa-miR-550a-3p | *hsa-miR-424-5p* | |
| hsa-miR-517a-3p | *hsa-miR-206* | |
| hsa-miR-517b-3p | *hsa-miR-145-5p* | |
| novel.269 | *hsa-miR-18a-3p* | |
| novel.111 | *hsa-miR-154-5p* | |
| novel.41 | *hsa-miR-152-3p* | |
| hsa-miR-6734-5p | *hsa-miR-199a-5p* | |
| hsa-miR-1247-5p | *hsa-miR-133a-3p* | |
| hsa-miR-195-5p | *hsa-miR-497-5p* | |
| novel.502 | *hsa-miR-455-3p* | |
| hsa-miR-4489 | *hsa-miR-199b-5p* | |
| hsa-miR-1323 | *hsa-miR-301a-3p* | |
| novel.301 | *hsa-miR-184* | |
| hsa-miR-6724-5p | *hsa-miR-205-3p* | |
| hsa-miR-885-5p | *hsa-let-7a-3p* | |
| novel.550 | *hsa-miR-29b-1-5p* | |
|  | *hsa-miR-411-5p* | |
|  | *hsa-let-7c-3p* | |
|  | *hsa-miR-299-3p* | |
|  | *hsa-miR-376a-3p* | |
|  | *hsa-miR-196a-5p* | |
|  | *hsa-miR-889-3p* | |
|  | *hsa-miR-376c-3p* | |
|  | *hsa-miR-195-3p* | |
|  | *hsa-miR-214-5p* | |
|  | *hsa-miR-3157-3p* | |
|  | *novel.80* | |
